# Demographic and Socioeconomic Factors Associated With ART Nonadherence Among 15 Years and Older South Africans. Evidence From the 2022 Cross‐Sectional Population‐Based Household Survey

**DOI:** 10.1155/arat/7225073

**Published:** 2026-05-23

**Authors:** Sbonelo Chamane, Musawenkosi Mabaso, Tarylee Reddy, Prudence Chambale, Kutloano Skhosana, Ronel Sewpaul, Lehlogonolo Makola, Angeline Ngcobo, Vuyelwa Mehlomakulu, Lesiba Molopa, Sean Jooste, Sizulu Moyo, Nompumelelo Zungu, Khangelani Zuma

**Affiliations:** ^1^ Public Health, Societies and Belonging Division, Human Sciences Research Council, Pretoria, South Africa, hsrc.ac.za; ^2^ School of Public Health, University of the Witwatersrand, Johannesburg, South Africa, wits.ac.za; ^3^ Biostatistics Unit, South African Medical Research Council, Durban, KwaZulu-Natal, South Africa, mrc.ac.za; ^4^ Information and Corporate Management, Durban University of Technology, Durban, South Africa, dut.ac.za; ^5^ School of Public Health and Family Medicine, University of Cape Town, Cape Town, South Africa, uct.ac.za; ^6^ School of Nursing and Public Health, University of KwaZulu-Natal, Durban, South Africa, ukzn.ac.za

**Keywords:** ART, HIV, nonadherence, PLHIV, South Africa

## Abstract

**Background:**

Globally, South Africa has the highest number of people living with HIV (PLHIV) and the largest HIV treatment programme. Adherence to antiretroviral therapy (ART) is a key factor in achieving viral load suppression and positive health outcomes and is, therefore, a crucial component in managing the HIV epidemic.

**Methods:**

The survey data were collected using a two‐stage stratified cluster random sampling design. Descriptive statistics were used to summarise the sample characteristics including the prevalence of nonadherence to ART. Pearson chi‐square was used to test for differences in categorical variables. Bivariate modified Poisson regression analysis was used to investigate factors associated with nonadherence to ART, and statistically significant variables were included in a multivariate modified Poisson regression model.

**Results:**

Of 3737 participants who self‐reported ever taking ART, 11.6% were classified as nonadherent (no antiretroviral [ARV] drugs detected in the dry blood spot). In the final model, participants with secondary or Grade 12 education had significantly higher prevalence of nonadherence than those with no or primary education (adjusted prevalence ratio [aPR] = 1.82; 95% CI: 1.12–2.95; *p* = 0.015). Decreased prevalence of ART nonadherence was associated with those aged 35–44 years (aPR = 0.48; 95% CI: 0.28–0.82; *p* = 0.007) and 55–64 years (aPR = 0.39; 95% CI: 0.18–0.84; *p* = 0.016) compared to those aged 15–24 years, and those residing in rural formal/farm areas compared to those living in urban areas (aPR = 0.27; 95% CI: 0.14–0.54; *p* < 0.001).

**Conclusion:**

The study highlights higher risk of nonadherence to ART among youth and those who reside in urban areas which could be improved through youth‐friendly interventions and ongoing tailored interventions for PLHIV in urban areas.

## 1. Background

In South Africa, HIV remains a major public health challenge, with an estimated 8 million people living with HIV (PLHIV) in 2022 [1]. The country also has one of the largest HIV treatment programmes globally, with an estimated 5.7 million people on antiretroviral therapy (ART) in 2022 [2–4]. Optimizing adherence to ART is a key strategy for both treatment and prevention of HIV. Measures of adherence to ART refers to the extent to which a person’s behaviour—taking medication, following a diet and/or executing lifestyle changes—corresponds with agreed recommendations from a healthcare provider [5, 6]. Adherence to ART is associated with viral suppression, improved health outcomes and interruption of transmission when viral load is undetectable [7, 8]. Nonadherence among PLHIV has public health ramifications such as increased morbidity and mortality and compromises the quality of life by increasing chances of treatment failure [9, 10].

Multiple individual‐level and system‐related factors can influence adherence including age, sex, support system, substance use, HIV stigma, knowledge and attitudes about ART, side effects from ART, food requirements and healthcare system‐related factors such as distance travelled to facility or availability of treatment (stock shortage) [11–13]. Evidence shows that advancements in ART and its delivery have improved adherence and are responsible for the HIV response’s successes [14–18]. However, several factors, including sociodemographic and socioeconomic disparities, such as differences in education, employment, place of residence and income, can significantly impact adherence to treatment [19].

Identifying factors associated with ART nonadherence is vital in the fight against the HIV epidemic in the country [20, 21]. Understanding these factors allows for targeted interventions to improve adherence and ultimately reduce HIV transmission and morbidity [22]. However, nonadherence to therapy can be difficult to measure accurately [23]. Most studies rely on data from self‐reports of missing pills, assays of drug levels, which have been used in clinical trials, and electronic monitoring systems [24, 25]. Secondly, there is a paucity of large‐scale population‐based studies of ART adherence. Accurate measurement of adherence is crucial in the assessment of the determinants of nonadherence and how it can be improved [26, 27].

This study examines demographic and socioeconomic factors associated with ART nonadherence measured through laboratory detection of antiretroviral (ARV) drugs, among individuals aged 15 years and older who self‐reported ever taking ART to treat HIV in the cross‐sectional population‐based household survey conducted in South Africa in 2022.

## 2. Materials and Methods

### 2.1. Design and Sampling

Data analysed was obtained from the sixth South African National HIV Prevalence, Incidence, Behaviour Survey (SABSSM VI), conducted in 2022 described in detail elsewhere [4]. The data used were collected using a cross‐sectional population‐based household survey conducted using a two‐stage stratified cluster random sampling design. The survey used a systematic random sample of 2114 small area layers (SALs) extracted from Statistics South Africa master sample of 84,907 SALs as the primary sampling unit. The secondary sampling unit was a systematic random sample of 15 visiting points (VPs) and households sampled from each of the 2114 SALs. These SALs were stratified by province and locality type (categorised by Statistics South Africa as urban, rural formal [farms] and rural informal [traditional tribal areas and rural villages]). The ultimate sampling unit was all individuals living in selected households. All individuals within selected households were eligible to participate.

### 2.2. Laboratory Testing

The questionnaires included in SABSSM VI collected information on HIV‐related knowledge, attitudes, practices, behaviours, and demographic information, including ART and treatment interruption. Dried blood spot (DBS) samples were collected from consenting individuals for testing to assess HIV serostatus, ARV drugs, viral suppression and HIV drug resistance and for limiting antigen avidity enzyme immunoassay testing as part of the survey’s HIV incidence estimation algorithm. The drugs included in the ARV testing panel were atazanavir, dolutegravir (DTG), efavirenz, lopinavir and nevirapine and were selected based on the ART regimens used in the South African public health sector at the time of the survey. Barcodes were used to link blood samples and test results to corresponding questionnaires.

### 2.3. Ethical Approval

The survey protocol was approved by the HSRC Research Ethics Committee (REC: 4/18/11/15), and the U.S. Centers for Disease Control and Prevention (CDC) reviewed the protocol in accordance with human research protection procedures and relied on the local REC for approval^1^.

### 2.4. Theoretical Framework

The factors influencing ART adherence can be viewed through the lens of the socioecological model [28, 29]. This theoretical framework emphasises that individual intentions, health decisions and behaviour do not occur in a vacuum but are influenced by several factors within a given context. This model recognises that individual, interpersonal, community and societal level factors influence health behaviour [30, 31]. Therefore, the model helps us to understand the multifaceted contextual factors within which PLHIV live and how their interaction influences ART adherence. Situating this study within the context of the socioecological model, the current analysis explores the extent to which ART adherence is influenced by individual‐level factors such as demographic and socioeconomic characteristics.

### 2.5. Statistical Measures

This study focuses on participants who self‐reported ever initiating ART and estimates nonadherence based on the presence or absence of ARV drugs in dried blood samples. The ARVs tested vary significantly in terms of their half‐lives: atazanavir (7 h), DTG (14 h), efavirenz (50 h), lopinavir (6 h), and nevirapine (57 h) [32–36]. Since it typically takes about 5 half‐lives as a maximum for a drug to be fully eliminated from the body [37], these correspond to elimination times of approximately 35 h (1.5 days) for atazanavir, 70 h (2.9 days) for DTG, 250 h (10.4 days) for efavirenz, 30 h (1.25 days) for lopinavir, and 285 h (11.9 days) for nevirapine. In this study, the absence of detectable ARV drugs was interpreted as indicative of not taking ART for approximately 1 to 12 days. If any of the tested drugs were detected, the participant was classified as adherent (coded as 0); if none were detected, they were classified as nonadherent (coded as 1).

The study included demographic and socioeconomic variables as exposure variables. The demographic variables included were sex of the respondent (female and male), age groups in years (15–24, 25–34, 35–44, 45–54, 55–64 and 65+), marital status (married and never married) and locality type (urban areas, rural informal/tribal areas and rural formal/farm areas). The socioeconomic measures used were employment status (employed and unemployed), highest education level (none/primary school, some secondary school/Grade 12 and tertiary education) and asset‐based socioeconomic status (SES) (high SES and low SES) constructed using multiple correspondence analyses (MCA) based on questions on availability/ownership of a broad range of household assets and utilities [38].

### 2.6. Statistical Analysis

The statistical analyses were performed using Stata statistical software Version 18.0 (Stata Corporation, College Station, USA). Descriptive statistics were used to summarise the sample characteristics, provide the prevalence of ART nonadherence and apply the Pearson’s chi‐square test of independence to examine the difference in the prevalence of nonadherence by sample characteristics. A bivariate modified Poisson regression was used to investigate factors associated with nonadherence to ART, and statistically significant variables were entered into a multivariate modified Poisson regression model. Adjusted prevalence ratio (aPR) and 95% confidence interval (CI), and *p* value ≤ 0.05 were used to determine the direction, strength of the association and level of statistical significance. The “svy” command was utilised in all analyses to account for the complex design.

## 3. Results

### 3.1. Sample Characteristics

**FIGURE 1:**
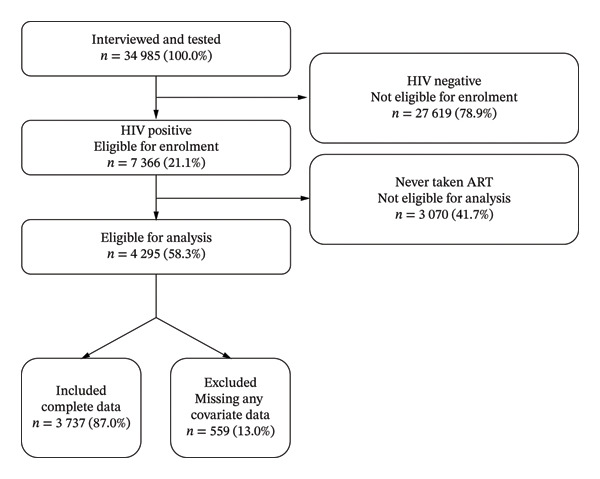
Flowchart of study participants showing inclusion, exclusion and analytic sample, 2022.

Out of 34,985 respondents who were interviewed and tested, 7366 (21.1%) HIV‐positive respondents aged 15 years and older were eligible for inclusion in the study. Of these, 4295 (58.3%) participants reported ever taking ART to treat HIV and had a valid ARV biomarker result, making them eligible for analysis. A total of 559 (13.0%) participants were excluded due to missing data on at least one independent variable. Therefore, the final study sample included 3737 (87.0%) participants who had ever taken ART to treat HIV (Figure 1).

The majority of the study sample were females (71.8%, Table 1), were aged 35–44 years (37.4%), were unemployed (72.7%), had completed some secondary school/Grade 12 education level (69.4%), were never married (75.4%), were from low SES households (57.3%) and resided in urban areas (60.0%).

**TABLE 1:** Distribution of the study sample aged 15 years and older who were HIV positive and had an ART status (*n* = 3737), South Africa, 2022.

Variables	*n*	Column (%)	95% CI
Sex of the respondent			
Male	910	28.2	25.0–31.7
Female	2827	71.8	68.3–75.0
Age groups in years			
15–24	218	4.3	3.3–5.6
25–34	833	23.8	20.4–27.6
35–44	1284	37.4	34.1–40.7
45–54	901	24.3	21.2–27.7
55–64	378	7.9	6.4–9.6
65+	123	2.3	1.4–3.9
Employment status			
Unemployed	2626	72.7	69.0–76.1
Employed	1111	27.3	23.9–31.0
Highest education level			
None/Primary school	960	22.0	19.4–24.8
Secondary school/Grade 12	2537	69.4	66.0–72.6
Tertiary education	240	8.6	6.5–11.4
Marital status			
Married	828	24.6	21.3–28.2
Never Married	2909	75.4	71.8–78.7
Asset‐based SES			
Low SES	2368	57.3	52.2–62.2
High SES	1369	42.7	37.8–47.8
Locality type			
Urban areas	2151	60.0	54.6–65.3
Rural informal/tribal areas	1050	32.8	27.8–38.3
Rural formal/farm areas	536	7.1	5.4–9.4

### 3.2. Prevalence of ART Nonadherence

Table 2 shows that of 3737 participants, 11.6% were nonadherent to ART. Nonadherence was significantly higher among participants residing in urban areas. Although not statistically significant, ART nonadherence was also higher among males, those aged 25–34 years, the unemployed, those who had tertiary education, those never married, and those from lower SES households.

**TABLE 2:** Prevalence of ART nonadherence among HIV‐positive participants aged 15 years and older, South Africa, 2022.

Variables	*n*	Nonadherence (%)	95% CI	*p*‐value^1^
Total	3737	11.6	8.2–16.1	
Sex of the respondent				0.566
Male	910	13.2	8.2–20.4	
Female	2827	11.0	7.0–16.9	
Age groups in years				0.071
15–24	218	17.4	11.6–25.4	
25–34	833	18.2	8.0–36.2	
35–44	1284	6.8	4.9–9.4	
45–54	901	13.8	8.2–22.4	
55–64	378	6.0	2.9–12.0	
65+	123	5.5	1.7–16.5	
Employment status				0.378
Unemployed	2626	12.5	8.4–18.3	
Employed	1111	9.2	5.1–16.0	
Highest education level				0.144
None/primary school	960	6.2	4.3–8.9	
Secondary school/Grade 12	2537	13.0	8.5–19.3	
Tertiary education	240	14.4	5.2–33.8	
Marital status				0.233
Married	828	8.3	4.6–14.4	
Never married	2909	12.7	8.5–18.6	
Asset‐based SES				0.764
Low SES	2368	12.1	7.1–19.6	
High SES	1369	11.0	7.8–15.3	
Locality type				0.022
Urban areas	2151	14.5	9.5–21.3	
Rural informal/tribal areas	1050	8.1	4.9–13.1	
Rural formal farm areas	536	3.6	1.9–7.0	

Abbreviation: CI, confidence interval.

^1^Pearson’s chi‐square test.

### 3.3. Factors Associated With ART Nonadherence

Factors found to be statistically significant in the bivariate modified Poisson regression included age group, highest education level, and locality (Table 3). These variables were subsequently included in the multivariate modified Poisson regression model. The multivariate analysis showed that the increased prevalence of ART nonadherence was significantly associated with participants with secondary or Grade 12 education compared to those with no or primary education [aPR = 1.87; 95% CI: 1.11–3.14; *p* = 0.018]. Decreased prevalence of ART nonadherence was significantly associated with participants aged 35–44 years [aPR = 0.42; 95% CI: 0.24–0.73; *p* = 0.002] and those aged 55–64 years [aPR = 0.42; 95% CI: 0.18–1.00; *p* = 0.049], compared to those aged 15–24 years. In addition, residing in rural formal or farm areas was significantly associated with decreased prevalence of ART nonadherence compared to those living in urban areas [aPR = 0.28; 95% CI: 0.14–0.57; *p* < 0.001].

**TABLE 3:** Modified Poisson regression of factors associated with ART nonadherence among HIV‐positive participants aged 15 years and older, South Africa, 2022.

Variables	Bivariate				Multivariate			
	PR	95% CI		*p*‐value	aPR	95% CI		*p*‐value
Sex of the respondent								
Male	Ref				—			
Female	0.83	0.43	1.60	0.581	—	—	—	—
Age groups in years								
15–24	Ref				Ref			
25–34	1.04	0.44	2.50	0.922	1.05	0.45	2.48	0.908
35–44	0.39	0.23	0.67	0.001	0.42	0.24	0.73	0.002
45–54	0.79	0.41	1.53	0.493	0.91	0.47	1.76	0.772
55–64	0.34	0.15	0.78	0.011	0.42	0.18	1.00	0.049
65+	0.31	0.09	1.07	0.064	0.48	0.13	1.75	0.266
Employment status								
Unemployed	Ref				—			
Employed	0.74	0.37	1.47	0.383	—	—	—	—
Highest education level								
None/Primary school	Ref				Ref			
Secondary school/Grade 12	2.08	1.19	3.62	0.010	1.87	1.11	3.14	0.018
Tertiary education	2.30	0.84	6.33	0.107	1.71	0.60	4.81	0.313
Marital status								
Married	Ref				—			
Never Married	1.53	0.76	3.11	0.236	—	—	—	—
Asset‐based SES								
Low SES	Ref				—			
High SES	0.91	0.48	1.72	0.774	—	—	—	—
Locality type								
Urban areas	Ref				Ref			
Rural informal (tribal areas)	0.56	0.29	1.06	0.075	0.60	0.34	1.05	0.076
Rural formal (farm areas)	0.25	0.12	0.53	< 0.001	0.28	0.14	0.57	< 0.001

Abbreviations: aPR, adjusted prevalence ratio; CI, confidence interval.

## 4. Discussion

This study examined the prevalence of ART nonadherence and the influence of demographic and socioeconomic factors among South Africans aged 15 years and older. ART nonadherence was highest among the youth aged 15–24 years and those aged 25–34 years. Globally, ART nonadherence has been reported to be more common among youth than older age groups [39–42]. In agreement with similar studies on adherence, we found that males were more nonadherent than females [43, 44], and this has been attributed to men’s general poor engagement with care and poor health seeking practices that are often associated with societal norms about what it means to be a man and stigma [10]. As observed in other studies, ART nonadherence was higher among the unemployed PLHIV compared to their counterparts [30, 31, 45]. This higher nonadherence among the unemployed is often linked to various socioeconomic factors, including poverty, lack of food, and financial insecurity, which can act as barriers to accessing and adhering to treatment [10, 46, 47]. In addition, ART nonadherence was higher among PLHIV with secondary school/grade 12 education compared to those with no/primary school education attainment. This may seem counterintuitive and contrary to other studies [31]. However, several other factors can contribute to ART nonadherence, even among individuals with higher education [10, 12]. Similarly, as observed elsewhere ART nonadherence was higher among PLHIV residing in low SES households than those in high SES households [48]. There is mixed evidence in the literature, with some studies reporting ART nonadherence among individuals with lower SES, while others found no association [19]. In agreement with other studies PLHIV residing in urban areas were more nonadherent than those in rural areas [49–51]. Although the proportions observed were higher across most categories, only locality type was significantly associated with ART nonadherence.

The final model confirmed the relationship between ART nonadherence and younger age. Evidence shows that youth, particularly adolescents and young adults aged 15–34 years, face distinctive social and behavioural challenges, such as peer pressure, stigma, and engagement in risky sexual behaviours, that can negatively affect ART adherence [52–54]. Other studies found that youth encounter discrimination in healthcare facilities, which discourages treatment engagement and interrupts continuity of care, leading to ART non‐adherence [20, 55]. In addition, structural health system barriers, such as long waiting times at primary healthcare (PHC) facilities where ART is dispensed, have been widely reported in South Africa and may further discourage consistent medication collection, particularly among younger individuals who may have competing educational, social or economic demands. These observations highlight the importance of age‐specific, youth‐friendly interventions, alongside health system strengthening strategies such as differentiated service delivery models, appointment spacing and fast‐track ART collection, to improve ART adherence.

The models also established that PLHIV with secondary school/Grade 12 educational attainment had a greater risk of ART nonadherence compared to those with no/primary school education. Studies have reported mixed results. One study on nonadherence found a positive correlation between higher education and ART nonadherence [56], implying that people with more education might be less likely to adhere to treatment. However, other studies have shown no significant relationship between education and adherence or even suggest that higher education is associated with better adherence [31, 57]. In addition to educational attainment, research on health literacy suggests that individuals with lower health literacy may be less likely to understand and follow treatment regimens, potentially impacting adherence [52].

Although HIV education has been formally integrated into the South African school curriculum through the Life Orientation programme since 2000, these findings highlight a critical gap between exposure to information and the development of actionable, sustained health literacy. This disconnect may reflect variability in the quality, depth and retention of HIV‐related content, as well as the timing of delivery relative to individuals’ lived experiences with HIV. In addition, school‐based education often focuses on prevention rather than long‐term treatment management, which may limit its effectiveness in supporting ART adherence in adulthood. Furthermore, successful ART adherence is a product of both individual discipline and external support systems, including both at the health facility and community levels [55, 57]. This underscores the need for continuous healthcare provider counselling to address treatment‐related barriers such as lack of motivation, side effects and forgetfulness, regardless of educational attainment. Equally important are structured social support systems, including treatment support groups, to encourage sustained ART adherence. These can be established within PHC facilities or communities using existing HIV programmes and facilitated by trained peer educators or counsellors. Monitoring can be integrated into routine systems through indicators such as attendance, retention in care and viral suppression.

The model also verified that ART nonadherence was associated with residing in urban areas. Other studies have reported the opposite, with urban residence being linked to better adherence [58]. However, low ART adherence has also been observed in PLHIV in low‐income urban and semiurban settings [59]. The higher ART nonadherence observed in urban areas may reflect comparatively limited adherence support structures in these settings. In contrast, rural areas often benefit from more targeted efforts—such as support provided by ward‐based outreach teams (WBOTs) and nongovernmental organizations (NGOs), which may enhance treatment adherence. This underscores the importance of developing contextually appropriate interventions to support ART adherence.

## 5. Limitations

The cross‐sectional nature of the study design does not allow for the establishment of a causal relationship between ART nonadherence and selected covariates. The missing ARV biomarker data among HIV‐positive participants may have introduced bias that cannot be assessed due to unavailable data. This study may be subject to residual confounding due to the exclusion of important factors known to influence ART adherence, including HIV‐related stigma, mental health status, substance use, social support, healthcare access barriers and treatment‐related factors. The absence of these variables may have affected the observed associations between sociodemographic characteristics and ART nonadherence. The study relied on self‐reported data, which may be subject response bias due to either social desirability bias and/or recall bias. As this was a secondary analysis of survey data, several key correlates of ART adherence such as regimen complexity, pill count and severity of side effects from the medications were not available for analysis. This survey was conducted during the initial rollout of DTG in South Africa; therefore, current adherence patterns may differ, given that the majority of PLHIV are now receiving ART as a single fixed‐dose combination (FDC) pill. Despite these limitations, the study was nationally representative, and therefore, the findings can be generalised to the South African population.

## 6. Conclusion

The study identified PLHIV at risk of ART nonadherence and suggested interventions that may improve adherence, particularly in at‐risk individuals such as younger people, those with secondary school/Grade 12 educational qualifications and those residing in urban areas. The findings suggest a need for age‐specific youth‐friendly interventions to improve ART adherence. Integrating comprehensive HIV education into the basic education school curriculum may improve HIV‐related health literacy, promote early treatment engagement and potentially enhance long‐term ART adherence. The findings also suggest a need for context‐sensitive interventions to support ART adherence among PLHIV in the country. These findings have direct implications for key stakeholders. The Department of Health can use these results to design and implement tailored ART adherence programmes, including youth‐friendly services and urban‐targeted interventions. The Department of Basic Education may incorporate comprehensive HIV education into school curricula, fostering early awareness and promoting adherence behaviours among adolescents. Community‐based organisations and NGOs involved in HIV care can leverage these insights to provide targeted support, adherence counselling and peer‐led programmes for high‐risk populations. Collectively, coordinated actions by these stakeholders can improve ART adherence, reduce HIV‐related morbidity and strengthen the national HIV response.

## Funding

The study was supported by the U.S. President’s Emergency Plan for AIDS Relief, NU2GGH002302.

## Conflicts of Interest

The authors declare no conflicts of interest.

## Endnotes

^1^See 45 C.F.R. part 46.114; 21 C.F.R. part 56.114.

## Data Availability

The data that support the findings of this study are available on request from the corresponding author. The data are not publicly available due to privacy or ethical restrictions.
